# Surplus of Women

**Published:** 1920-02-21

**Authors:** 


### Surplus of Women.

An interesting practical proposal and offer are made in a letter to the Press by the Agent-General for West Australia, who groups together for the purposes of his offer the fact of the surplus women population of Britain and the question of child welfare. He does not take count of the circumstance that infant welfare work, if it is scientifically intensified, would contain the possibilities of redressing the balance for the reason that, as more boy babies than girl babies are born (though fewer survive) the more infant welfare work succeeds the more will boy babies be saved. His main point, on the contrary, is that it is a pity the magnificent work of protecting child life should have no better future for a large proportion of children than that of swelling the ranks of "unwanted women"—not too pretty an expression.

### In the Dominions.

But he makes proposals which, however, stand by themselves and do not closely affect the foregoing points. He says the disparity of sexes is not so great in the Dominions as in this country, and that even such disparity as does exist is generally shown in a surplus of males While, therefore, he would not suggest anything like a wholesale migration of women and children to the Dominions, at least until more settled conditions obtain there, and while he gratefully acknowledges the good work that has been done by many societies in promoting the emigration of women and children, he thinks something more might be done in that direction.

"I am entitled to speak only for my own State—Western Australia—but, I have no doubt that most of the oversea Dominions could and would absorb a much larger number of immigrants of this class than has been possible with the present dearth of shipping. Surely it is not too much to ask the Ministry of Shipping and the Ministry of Transport to come to our assistance in this matter? It is not for the benefit of the Dominions alone; it is for the advantage of this country, which is suffering from over-population, and for the advantage of the Empire, which is suffering from an unequally distributed population.

"We in Western Australia cannot absorb immigrants in large numbers, but I am authorised by my Government to say that, as a beginning, we will take 200 war orphans between nine and fourteen years of age; 500 war orphans between fourteen and sixteen years of age; war widows, with or without children, and an unlimited number of domestic servants and farm workers.

"The Oversea Settlement Committee of the Colonial Office will give the war widows and their families and the war orphans free passages, and my Government will provide assisted passages for the domestics and country workers."

